# Case Report: Corticosteroid-induced severe psychiatric manifestations in a patient with leptomeningeal metastases from lung adenocarcinoma

**DOI:** 10.3389/fphar.2026.1939579

**Published:** 2026-09-16

**Authors:** Fan Yang, Lihong Guo, Xiangwen Xiao, Min Tang, Jun Meng

**Affiliations:** 1 Department of Pharmacy, National Cancer Center, National Clinical Research Center for Cancer, Cancer Hospital & Shenzhen Hospital, Chinese Academy of Medical Sciences and Peking Union Medical College, Shenzhen, China; 2 Department of Medicine-Oncology, National Cancer Center, National Clinical Research Center for Cancer, Cancer Hospital & Shenzhen Hospital, Chinese Academy of Medical Sciences and Peking Union Medical College, Shenzhen, China; 3 School of Pharmacy, Guangdong Medical University, Dongguan, China

**Keywords:** case report, clinical pharmacist, corticosteroid, leptomeningeal metastases, sensitization, severe psychiatric manifestations

## Abstract

**Background:**

Glucocorticoids are widely used in leptomeningeal metastases (LM) to reduce peritumoral edema, yet their psychiatric adverse effects remain underrecognized, particularly progressive sensitization following long-term tolerance.

**Case presentation:**

A 66-year-old male with EGFR-mutant lung adenocarcinoma and LM developed progressively worsening psychiatric manifestations after three short-course glucocorticoid exposures, despite >9 months of prior tolerance to intrathecal dexamethasone. Symptoms escalated from manic-like agitation to homicidal ideation to persecutory delusions with complete treatment refusal. A Naranjo score of 10 confirmed definite causality. Symptoms resolved after discontinuation and psychiatric hospitalization.

**Conclusion:**

This case demonstrates cumulative sensitization with paradoxical dose-independent worsening of corticosteroid-induced psychiatric toxicity following long-term tolerance, whereby repeated glucocorticoid exposures produced progressively severe psychiatric reactions despite fluctuating and overall decreasing cumulative doses, supporting a “basal vulnerability plus acute trigger” model and highlighting the pivotal role of clinical pharmacists in pharmacovigilance.

## 1 Introduction

Leptomeningeal metastasis (LM) is a devastating complication of advanced lung cancer, associated with poor prognosis and significantly impaired quality of life (Le Rhun et al., 2023; Hong et al., 2025). The 2025 Chinese Expert Consensus on Leptomeningeal Metastases of Lung Cancer, developed by the Neurological Tumor Specialist Committee of the Chinese Society of Clinical Oncology (CSCO), provides comprehensive guidance on diagnosis, risk stratification, and treatment, with intrathecal chemotherapy and targeted therapy as recommended treatment modalities (Lin et al., 2025).

Glucocorticoids are commonly used in patients with brain metastases and LM to reduce vasogenic edema and alleviate symptoms of elevated intracranial pressure, including headache, nausea, and vomiting. For symptomatic patients with brain metastases, recommended dexamethasone doses range from 4 to 8 mg daily for mild symptoms to ≥16 mg daily for moderate-to-severe mass effect (Ryken et al., 2010). However, glucocorticoid use is associated with a spectrum of neuropsychiatric adverse effects, ranging from emotional lability and insomnia to mania, psychosis, and violent behavior (Warrington and Bostwick, 2006).

A recent systematic review (Gostoli et al., 2025) reported that among 34 clinical cases of corticosteroid-induced mania and/or psychotic symptoms, 64.7% presented with both manic and psychotic features, 23.5% with psychotic symptoms only, and 11.8% with manic symptoms only. Meta-analyses show that behavioral changes occur in up to 52% of glucocorticoid users, with manic symptoms occurring in approximately 11% (Koning et al., 2024). Consistently, a prospective observational study reported psychiatric symptoms in up to 30% of corticosteroid users, with manic/hypomanic symptoms in 3.25% (Mukherjee and Roy, 2026). Risk factors for severe psychiatric reactions include prolonged courses, high-dose therapy, prior psychiatric history, advanced age, and female sex. Symptoms can emerge within 3–4 days of initiation. The underlying mechanisms involve glucocorticoid-receptor overstimulation, mineralocorticoid-receptor inactivation, and dysregulation of serotonin and dopamine systems (Nasereddin et al., 2024; Sofia-Avendano-Lopez et al., 2024). However, the phenomenon of progressive sensitization, in which psychiatric reactions worsen with repeated exposures despite prior tolerance, has rarely been documented in the context of LM. We report a patient with LM from lung adenocarcinoma who developed severe, progressively worsening psychiatric manifestations following three consecutive glucocorticoid exposures after long-term intrathecal tolerance, and discuss the role of the clinical pharmacist in identifying the adverse reaction and optimizing management.

## 2 Case presentation

### 2.1 Patient information and history

A 66-year-old male was diagnosed with stage IV lung adenocarcinoma (EGFR 19DEL+) in December 2023, with brain and widespread bone metastases. He had no prior psychiatric history and no family history of psychiatric disorders. From diagnosis until early 2025, he received almonertinib 110 mg daily combined with denosumab 120 mg every 4 weeks, with relatively stable disease. Between June and September 2024, he also underwent radiotherapy for brain, thoracic, and pelvic bone metastases.

### 2.2 Diagnostic assessment and timeline

In February 2025, the patient developed progressive dizziness, headache, gait instability, and lower-limb weakness. Brain MRI revealed stable parenchymal metastases (max 0.7 cm) but increased linear enhancement in the bilateral frontoparieto-occipital sulci (Figure 1A), consistent with LM. Cerebrospinal fluid (CSF) cytology confirmed meningeal involvement. Targeted therapy was switched to zorifertinib 200 mg twice daily combined with intrathecal chemotherapy via lumbar puncture (dexamethasone 5 mg, cytarabine 0.05 g, and methotrexate 10 mg every 28 days). One month later, the patient experienced a single generalized tonic-clonic seizure and thereafter received levetiracetam 0.5 g twice daily without recurrence. Over a 9-month period from February 2025 to October 2025, the patient tolerated regular intrathecal dexamethasone (5 mg per cycle) without any psychiatric symptoms. In July 2025, the regimen was adjusted to a weekly alternating schedule of almonertinib and zorifertinib.

**FIGURE 1:**
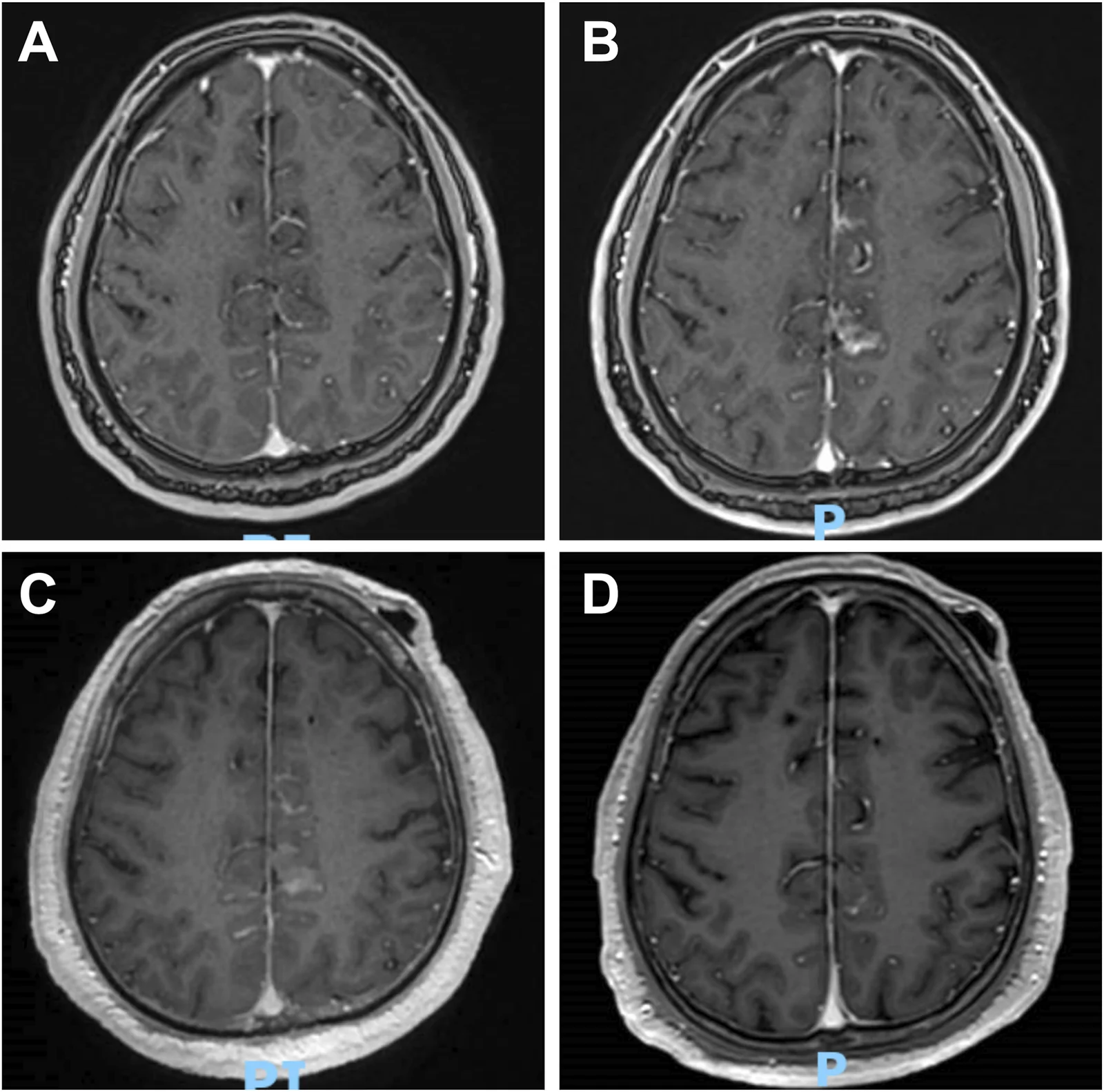
Serial brain MRI of a 66-year-old male with stage IV EGFR-mutant lung adenocarcinoma and brain metastases, showing the emergence and evolution of leptomeningeal enhancement over the clinical course. **(A)** 14 February 2025: linear enhancement is visible along the bilateral frontoparieto-occipital sulci, while the parenchymal metastases remained stable (maximum diameter 0.7 cm); these findings were consistent with leptomeningeal metastases, as subsequently confirmed by cerebrospinal fluid cytology, and prompted a switch of targeted therapy to zorifertinib combined with intrathecal chemotherapy. **(B)** 10 November 2025: persistent leptomeningeal enhancement along the interhemispheric fissure and cerebral sulci during the period of disease progression that necessitated further adjustment of the targeted therapy regimen. **(C)** 23 December 2025: marked reduction in both the extent and intensity of the meningeal enhancement compared with the previous scan, indicating a favorable response to treatment and arguing against disease progression as an explanation for the concurrent psychiatric symptoms. **(D)** 25 February 2026: further interval improvement of the meningeal enhancement following glucocorticoid discontinuation and continued antineoplastic therapy, corroborating sustained disease control and supporting the exclusion of tumor progression in the differential diagnosis of the patient’s psychiatric manifestations.

### 2.3 Three glucocorticoid exposures and psychiatric manifestations

Clinical details of the three glucocorticoid exposures, including regimens, cumulative prednisone equivalents, psychiatric manifestations, interventions, and outcomes, are summarized in Table 1.

**TABLE 1:** Summary of clinical characteristics and neuropsychiatric manifestations across three episodes of glucocorticoid exposure in the patient.

Exposure	Dates	Glucocorticoid regimen	Cumulative prednisone equivalent*	Symptom onset	Main psychiatric manifestations	Psychiatric interventions	Outcome
First exposure	28 Nov – 2 Dec 2025	Methylprednisolone 80 mg i.v. (Day 1–4) + 40 mg i.v. (Day 5)	∼450 mg	36 h after initiation	Euphoria, pressured speech, agitation, attempts to remove catheter	Olanzapine 5 mg once	Partially controlled
Second exposure	9–11 Dec, 2025	Dexamethasone 5 mg i.v. (Day 1) + methylprednisolone 40 mg i.v. (Day 3)	∼83 mg	24–36 h after initiation	Severe agitation, disorganized speech, homicidal ideation, tearing clothing	Olanzapine escalated to 5 mg bid from 12 December (clinical pharmacist recommendation); multiple doses of i.v. diazepam (10 mg); adjusted to 5 mg at noon +10 mg at bedtime (15 mg/day) with clonazepam 0.5 mg at bedtime by 18 December; psychiatry consultation on 23 December, after which clonazepam was switched to alprazolam 0.4 mg at bedtime and magnesium valproate 0.25 g twice daily was added	Gradual improvement, incomplete resolution
Third exposure	30 Dec 2025 – 1 Jan 2026	Dexamethasone 3.75 mg bid p.o. (Day 1–3) + 10 mg i.v. (Day 1) + 5 mg i.t. (Day 2)	∼250 mg	36 h after initiation	Persecutory delusions, paranoid beliefs, complete treatment refusal, discontinuation of all medications, refusal of food/water	Transfer to psychiatric hospital; continued olanzapine and magnesium valproate	Significant resolution within 10 days after glucocorticoid discontinuation

* Prednisone equivalent: dexamethasone ×6.67; methylprednisolone ×1.25.

i.v., intravenous; p.o., oral; i.t., intrathecal.

#### 2.3.1 First exposure

On 13 November 2025, the patient underwent left ventricular Ommaya reservoir placement and right ventriculoperitoneal shunt to facilitate subsequent intrathecal chemotherapy and relieve intracranial hypertension. Postoperative CSF culture grew *Enterobacter aerogenes*, and meropenem was initiated. From 28 November to 2 December 2025 (postoperative days 16–20), the patient received intravenous methylprednisolone 80 mg on Day 1–4, then 40 mg on Day 5 (cumulative prednisone-equivalent ≈450 mg). Thirty-six hours after glucocorticoid initiation, the patient developed marked euphoria, pressured speech, agitation, and repeated attempts to remove his intravenous catheter. Olanzapine 5 mg was administered once. The clinical pharmacist recommended early discontinuation after Day 3, but the attending physician opted to complete the planned 5-day course.

#### 2.3.2 Second exposure

On 9 December 2025, the patient underwent shunt removal due to persistent *Enterobacter aerogenes* infection with high fever that could not be effectively controlled by meropenem antimicrobial therapy. Glucocorticoids were given on two separate days within a 3-day period (dexamethasone 5 mg on Day 1 and methylprednisolone 40 mg on Day 3; cumulative prednisone-equivalent ≈83 mg). Severe agitation, disorganized speech, and homicidal ideation ensued, including tearing of clothing and the intravenous line. On 12 December, the clinical pharmacist recommended increasing olanzapine to 5 mg twice daily, with multiple doses of intravenous diazepam 10 mg administered. By 18 December, olanzapine was further adjusted to 5 mg at noon and 10 mg at bedtime (15 mg daily), with clonazepam 0.5 mg at bedtime. A psychiatry consultation was obtained on 23 December, after which clonazepam was switched to alprazolam 0.4 mg at bedtime, and magnesium valproate 0.25 g twice daily was added. Psychiatric symptoms gradually improved but did not resolve completely. Brain MRI on 23 December (Figure 1C) demonstrated further reduction in meningeal enhancement compared with the previous scan on 10 November (Figure 1B), excluding disease progression.

#### 2.3.3 Third exposure

From 30 December 2025 to 1 January 2026, the patient received glucocorticoids for three consecutive days in accordance with the pemetrexed premedication protocol, whereby dexamethasone is administered the day before, on the day of, and the day after pemetrexed infusion to reduce the incidence and severity of skin reactions (oral dexamethasone 3.75 mg twice daily plus intravenous 10 mg, followed by intrathecal dexamethasone 5 mg; cumulative prednisone-equivalent ≈250 mg). Psychiatric symptoms worsened dramatically, with persistent agitation, persecutory delusions, paranoid beliefs, and complete refusal of all treatments. The clinical pharmacist again recommended immediate glucocorticoid discontinuation; the attending physician adopted the recommendation this time.

### 2.4 Therapeutic intervention and follow-up

Following glucocorticoid discontinuation on 1 January 2026, the patient remained hospitalized with ongoing olanzapine and magnesium valproate, but psychiatric symptoms persisted unabated, necessitating transfer to a psychiatric hospital on 5 January. After approximately 10 days of inpatient treatment, psychiatric symptoms resolved significantly. He was discharged on 14 January 2026, continuing olanzapine 15 mg daily and magnesium valproate 0.25 g twice daily. Follow-up brain MRI on 25 February 2026 demonstrated improved meningeal enhancement (Figure 1D). Subsequent intrathecal pemetrexed cycles were administered without glucocorticoids, with stable disease; however, skin rash exacerbated markedly following intrathecal administration. A reduced glucocorticoid dose (dexamethasone 4 mg orally) was later used successfully to control skin reactions.

### 2.5 Concomitant medications

Throughout the treatment course, the patient received meropenem 2 g every 8 h (14 November–3 December, and 5–29 December, 2025); zorifertinib (discontinued 13–20 November, 2025, and resumed on 28 November in combination with almonertinib owing to disease progression); and the various psychiatric medications as described. No other drugs known to frequently induce psychosis (e.g., interferons, antimalarials, dopaminergic agents) were administered.

### 2.6 Patient perspective

The patient’s family reported that the unpredictable psychiatric episodes were more distressing than physical symptoms; after glucocorticoid discontinuation, the patient regained meaningful engagement, and both family and patient expressed gratitude to the clinical pharmacist.

## 3 Discussion

### 3.1 Dose–response and cumulative nature of psychiatric toxicity: a sensitization phenomenon

The most striking observation is the progressive worsening of psychiatric symptoms with each successive glucocorticoid exposure, despite decreasing cumulative doses. The patient tolerated long-term low-dose intrathecal dexamethasone for over 9 months but developed escalating reactions to acute systemic exposures. This pattern is consistent with a sensitization or kindling phenomenon. The first exposure induced manic-like symptoms partially controlled by olanzapine; the second exposure triggered severe agitation and homicidal ideation; the third exposure induced persecutory delusions and complete treatment refusal, necessitating psychiatric hospitalization.

This phenomenon has been described in the literature, though mechanisms remain incompletely understood (Warrington and Bostwick, 2006). Repeated exposures may lead to cumulative dysregulation of glucocorticoid- and mineralocorticoid-receptor balance, as mechanistically detailed in Section 3.2. Moreover, recurrent infections, surgical stress, and targeted therapy changes may have lowered the threshold for psychiatric symptoms. The key clinical message is that prior tolerance does not guarantee future safety. Specifically, repeated exposures, even at lower doses, can produce progressively worsening reactions.

### 3.2 Pharmacological mechanisms

Glucocorticoids affect central nervous system function through both genomic and non-genomic pathways. They can induce excitotoxic changes in hippocampal and striatal neurons and increase cerebral inflammation and oxidative stress (Sofia-Avendano-Lopez et al., 2024). The hippocampus, critical for emotional and memory regulation, is particularly sensitive to glucocorticoid damage. Glucocorticoids also modulate the hypothalamic-pituitary-adrenal axis, influencing dopamine, serotonin, and glutamate levels. Dopaminergic overactivity via mesolimbic pathways is a core mechanism of psychotic symptoms, and glucocorticoids may induce NMDA-receptor-mediated excitotoxicity (Nasereddin et al., 2024).

Glucocorticoid-induced psychiatric symptoms follow a U-shaped concentration–response curve: physiological levels are protective, but high levels are neurotoxic. The sensitization observed in this case was characterized by tolerance to 9 months of low-dose intrathecal dexamethasone followed by severe reactions to acute systemic exposures. This phenomenon reflects a central paradox of the present case: psychiatric toxicity escalated despite more than 9 months of uneventful tolerance to intrathecal dexamethasone. This paradox can be systematically explained by the interplay of three complementary sensitization mechanisms. First, subclinical hippocampal injury from long-term intrathecal glucocorticoid exposure: although the repeated low-dose intrathecal dexamethasone (5 mg per cycle) produced no overt psychiatric symptoms, chronic glucocorticoid exposure is known to induce excitotoxic and oxidative changes in hippocampal neurons, as noted above. Such cumulative, clinically silent injury may have created a “primed” state in which limbic circuits operated at the margin of functional compensation. Second, discrepancies in blood–brain barrier permeability and route-dependent pharmacokinetics: intrathecal administration achieves high local cerebrospinal fluid concentrations with limited systemic distribution, whereas subsequent intravenous and oral exposures generate systemic peak concentrations with different blood–brain barrier penetration profiles and abrupt central nervous system receptor engagement. The switch of route may thus have converted a tolerated exposure pattern into an effectively novel pharmacological challenge. Third, adaptive remodeling of glucocorticoid receptors: chronic exposure can induce adaptive changes in glucocorticoid- and mineralocorticoid-receptor expression and balance, such that the receptor system recalibrates to a state that paradoxically increases vulnerability upon re-exposure at different doses or by different routes.

Taken together, these three mechanisms converge on a coherent interpretation of the observed paradox: prior tolerance reflected a compensated equilibrium maintained by adaptive receptor remodeling under a stable intrathecal regimen, whereas each acute systemic exposure disrupted this equilibrium, delivering abrupt central receptor stimulation to hippocampal and limbic circuits already “primed” by subclinical injury, and thereby produced progressively severe psychiatric reactions even as cumulative doses decreased. This mechanistic framework operationalizes the “basal vulnerability plus acute trigger” model discussed in Section 3.3 and explains why prior tolerance did not guarantee future safety.

### 3.3 Modifying effect of leptomeningeal metastasis on psychiatric toxicity

LM itself can affect mental function through direct tumor invasion, intracranial hypertension, cerebral edema, and tumor-related seizures. The LM-induced microenvironmental changes in prefrontal and temporal cortex regions may have lowered the patient’s threshold for glucocorticoid psychiatric toxicity. When glucocorticoids further exacerbate neurotransmitter imbalances, brain regions already at the margin of functional compensation were more prone to decompensation. This “basal vulnerability plus acute trigger” dual-hit model, underpinned by the three sensitization mechanisms integrated in Section 3.2, may explain why the same glucocorticoid dose can produce markedly different psychiatric responses across patients, and why this patient decompensated so severely despite prior tolerance.

### 3.4 Differential diagnosis

Disease progression: Brain MRI on 25 February 2026, showed improved meningeal enhancement (Figure 1D), making progression unlikely.

Infection: With symptoms emerging 24–36 h after each glucocorticoid exposure, this temporal relationship strongly implicates glucocorticoids rather than the meropenem-treated intracranial infection.

Paraneoplastic limbic encephalitis: Inconsistent with the episodic, exposure-linked pattern and resolution upon discontinuation.

Primary psychiatric disorder: The patient had no prior psychiatric history, and symptoms resolved upon drug withdrawal, consistent with DSM-5-TR criteria for substance/medication-induced psychotic disorder (American Psychiatric Association, 2022). Glucocorticoids are explicitly listed as a cause of transient psychotic syndromes.

### 3.5 Naranjo causality assessment

The Naranjo Adverse Drug Reaction Probability Scale was applied. Based on the 10 standard questions: (1) previous conclusive reports of corticosteroid-induced psychosis (+1); (2) adverse event appeared within 24–36 h after each exposure (+2); (3) improvement upon discontinuation (+1); (4) recurrence and worsening upon re-exposure (+2); (5) no reasonable alternative causes (brain MRI stable, no primary psychiatric history, infection excluded) (+2); (6) placebo rechallenge not performed (0); (7) drug toxic levels not measured (0); (8) dose-response relationship was atypical due to sensitization (0); (9) similar reactions occurred after previous exposures (+1); (10) objective evidence including psychiatric consultation records and brain MRI (+1). The total score was 10 (≥9 = “Definite”), confirming definite causality (Naranjo et al., 1981).

### 3.6 Pharmacological management

Management of acute steroid-induced psychiatric symptoms follows a stepwise approach: taper or discontinue corticosteroids if clinically feasible, use antipsychotics and/or benzodiazepines for symptomatic treatment, and consult psychiatry for severe or refractory cases (Dubovsky et al., 2012).

Olanzapine was chosen for its dual dopamine D2 and serotonin 5-HT2A antagonism. However, in steroid-induced psychosis, excessive 5-HT2A blockade can paradoxically worsen manic symptoms via indirect prefrontal dopamine disinhibition (Schoenfeld et al., 2022). Benzodiazepines with serotonergic agonist properties, such as clonazepam, may be effective in such situations. The addition of magnesium valproate as a mood stabilizer likely provided adjunctive anti-impulsive effects. The delayed psychiatry consultation (obtained on 23 December, approximately 11–13 days after symptom onset) underscores the need for earlier involvement.

### 3.7 Role of the clinical pharmacist

This case highlights the critical role of clinical pharmacists in pharmacovigilance. The clinical pharmacist identified the temporal relationship after the first exposure, persisted in recommending discontinuation through subsequent exposures, and ultimately facilitated a management shift after the third exposure. After symptom resolution, the pharmacist recommended a reduced glucocorticoid dose (dexamethasone 4 mg orally) to manage skin reactions, demonstrating safe reintroduction when clinically necessary. This case supports the integration of clinical pharmacists into neuro-oncology multidisciplinary teams to monitor for delayed or sensitized adverse drug reactions.

### 3.8 Balancing glucocorticoid benefits and risks

Glucocorticoids remain valuable for managing cerebral edema in LM (Hong et al., 2025), but their neuropsychiatric adverse effects are not uncommon, as summarized in the Introduction. Clinicians should use the lowest effective dose for the shortest necessary duration, monitor for psychiatric symptoms daily, involve clinical pharmacists in medication review, and reassess the glucocorticoid indication daily. In patients with prior long-term intrathecal exposure, even brief systemic courses require close monitoring for paradoxical sensitization.

### 3.9 Study limitations

This is a single case report, and findings may not be generalizable. Concomitant medications (antibiotics, targeted agents) may have contributed to or modified the manifestations, although the consistent temporal relationship with glucocorticoid exposures makes glucocorticoids the most likely culprit. No formal psychiatric rating scales were used. Genetic testing for hypothalamic-pituitary-adrenal axis-related polymorphisms was not performed.

## 4 Conclusion

We present a case of severe, progressively worsening corticosteroid-induced psychiatric manifestations following three consecutive glucocorticoid exposures in a patient with LM, despite over 9 months of prior tolerance to intrathecal dexamethasone. Symptoms resolved after glucocorticoid discontinuation and psychiatric inpatient treatment. A Naranjo score of 10 confirms definite causality. This case demonstrates cumulative sensitization with paradoxical dose-independent worsening of glucocorticoid psychiatric toxicity following long-term tolerance, whereby repeated glucocorticoid exposures produced progressively severe psychiatric reactions despite fluctuating and overall decreasing cumulative doses, supporting the “basal vulnerability plus acute trigger” model. Key clinical messages include: (1) prior tolerance does not guarantee safety; (2) early recognition, timely discontinuation, and multidisciplinary collaboration are essential; (3) clinical pharmacists play a pivotal role; and (4) when glucocorticoids are necessary, the lowest effective dose for the shortest duration should be used with daily psychiatric monitoring.

## Data Availability

The original contributions presented in the study are included in the article, further inquiries can be directed to the corresponding author.
